# Association between neighborhood socioeconomic status and screen time among pre-school children: a cross-sectional study

**DOI:** 10.1186/1471-2458-10-367

**Published:** 2010-06-24

**Authors:** Valerie Carson, John C Spence, Nicoleta Cutumisu, Lindsey Cargill

**Affiliations:** 1 Faculty of Physical Education and Recreation, E-488 Van Vliet Center, University of Alberta, Edmonton, AB, Canada, T6G 2H9

## Abstract

**Background:**

Sedentary behavior is considered a separate construct from physical activity and engaging in sedentary behaviors results in health effects independent of physical activity levels. A major source of sedentary behavior in children is time spent viewing TV or movies, playing video games, and using computers. To date no study has examined the impact of neighborhood socioeconomic status (SES) on pre-school children's screen time behavior.

**Methods:**

Proxy reports of weekday and weekend screen time (TV/movies, video games, and computer use) were completed by 1633 parents on their 4-5 year-old children in Edmonton, Alberta between November, 2005 and August, 2007. Postal codes were used to classified neighborhoods into low, medium or high SES. Multiple linear and logistic regression models were conducted to examine relationships between screen time and neighborhood SES.

**Results:**

Girls living in low SES neighborhoods engaged in significantly more weekly overall screen time and TV/movie minutes compared to girls living in high SES neighborhoods. The same relationship was not observed in boys. Children living in low SES neighborhoods were significantly more likely to be video game users and less likely to be computer users compared to children living in high SES neighborhoods. Also, children living in medium SES neighborhoods were significantly less likely to be computer users compared to children living in high SES neighborhoods.

**Conclusions:**

Some consideration should be given to providing alternative activity opportunities for children, especially girls who live in lower SES neighborhoods. Also, future research should continue to investigate the independent effects of neighborhood SES on screen time as well as the potential mediating variables for this relationship.

## Background

Sedentary behavior is considered a separate construct from physical activity [1-5] and engaging in sedentary behaviors affects health independent of physical activity [5]. Therefore, the determinants of these behaviors should also be considered separately [3]. A major source of sedentary behavior in children is time spent viewing TV or movies, playing video games, and using computers [6]. Professional pediatric organizations recommend that children do not engage in more than 1-2 hours of screen time daily [7,8]. For pre-school children, the Canadian Pediatric Association recommends less than 1 hour a day [8]. However, The Health Behavior in School-Aged Children Survey (HBSC) revealed that 82% of Canadian girls and 86% of Canadian boys in grades 6 to 10 are not meeting the 2-hours per day guidelines [9].

These findings are of concern as a recent longitudinal cohort study found that viewing TV more than 2 hours per day as a child and adolescent was associated with overweight, poor fitness, increased smoking, and elevated cholesterol in adulthood [10]. This could be explained by several mechanisms including the lowering of children's metabolic rate [11,12], encouragement of between meal snacking, [13,14] or exposure to advertisements for junk food [14-16]. Therefore, it is critical to understand the determinants of screen time. However, unlike physical activity, little is known about the determinants of screen time [5,17-19]. Parental socioeconomic status (SES), ethnicity, body weight, between meal snacking, number of parents in the home, parents TV viewing habits, weekend, and having a TV in one's bedroom have been linked to TV viewing in children and adolescents between the ages of 2 and 18 [17]. Recently, characteristics of neighborhood environment (e.g., safety, sidewalk characteristics, walkability, income) have also been examined as determinants of screen time in children, adolescents, [20-22] and adults [23]. For example, MacLeod and colleagues found girls living in lower income neighborhoods in the United States watched more TV compared to girls living in higher income areas independent of family SES [22]. It is possible that children living in lower income neighborhoods engage in more screen time compared to children living in higher income neighborhoods because there are less resources for after-school programs [24] and recreational facilities in these neighborhoods [22]. Also, perceived lack of safety in lower income neighborhoods may limit children's outdoor play and increase sedentary indoor activity such as screen time [20,22,25,26].

Though aspects of the neighborhood environment have been associated with screen time among older children, to date no study has examined the association of neighborhood SES with pre-school children's screen time behavior. Since increased screen time engagement in early childhood is associated with increased engagement in school age children [27] it is especially important to understand the factors that influence screen time among younger children. This will inform preventive interventions aimed at reducing the adverse health effects of screen time that have been well documented among older children and adults [27]. Therefore, the main purpose of this study was to determine whether neighborhood SES is associated with screen time use among, 4- and 5- year-old boys and girls.

## Methods

### Participants

Children who were attending a Capital Health Center for preschool immunization in the Edmonton region between November 2005 and August 2007 were recruited for a longitudinal cohort study to investigate the determinants of childhood obesity. The city of Edmonton is the capital of the Province of Alberta and is located in western Canada. It is the largest northern city in North America with a metropolitan population of 1,034,045 in 2006 [28]. The data reported here is from the baseline phase of that study. Though not mandatory, a high proportion of children in the region visit these health Centers for immunizations and other services from birth through to preschool. Approximately, 74% of children were immunized for DTap-PIV (Diphtheria, Tetanus, Pertussis, and Polio) in the Capital Health region before entering grade 1 in 2004 [29]. Therefore, these health Centers allow access to the majority of children in the region. In total, 2114 parents participated with their 4- or 5- year-old child. Of those children, 1633 (805 girls and 828 boys) were part of the analyses; those excluded were 391 whose parent's did not complete the physical activity portion of the questionnaire correctly and 90 cases lacking age, BMI, SES, or daycare information.

### Procedures

For a child to receive his or her immunization booster shot, their parent had to schedule an appointment with a Capital Health Center. Once an appointment was booked, the parents were contacted by mail and asked if they would be interested in participating in the study. Those parents who were interested were then contacted prior to their appointment by telephone. The study was then explained to the parents and any questions were answered. If the parents were still interested they were mailed an information letter, consent form, and brief questionnaire, which they were to bring to their child's appointment. If parents forgot to bring their package to the appointment, extra copies were available at the health center. The questionnaire required approximately 20 minutes to complete and included questions on their child's food and beverage consumption, eating behaviors, physical activity, and screen time. Due to time restraints of the immunization appointments, no information was collected about the parent.

### Instruments

#### Screen Time

Children's screen time was assessed through a proxy report on leisure activities completed by a parent. It consisted of a checklist of 4 leisure activities including: TV/movies, playstation/nintendo/x-box/gameboy, computer/internet/computer games, and play indoors with toys. Parents indicated yes or no if their child participated in these activities in a typical week. For the activities circled "yes", parents recorded the total hours/minutes (duration) their child participated in the activity during Monday to Friday and/or Saturday and Sunday. At the end of the questionnaire parents could add other leisure activities their child participated in during a typical week along with the duration of those activities. Weighted means for weekdays and weekends were used to calculate the total weekly screen time minutes (TV/movies, video games, and computer use) for each participant.

#### Neighborhood Socioeconomic Status (SES)

Though children's addresses were not available, their postal codes were recorded in community health records. These postal codes were geocoded (assigned spatial reference) using the Postal Code Conversion File (PCCF) produced by Statistics Canada. An SES index was then created for each dissemination area where the centroids of children's postal codes were located using data extracted from the 2006 Census [30]. Dissemination areas are geographic units consisting of one or more adjacent blocks encompassing a population of 400 to 700 persons [31]. The SES index for each dissemination area was calculated by taking the sum of the z-scores of net educational level (the proportion of people with low education subtracted from the proportion of people with high education aged 15 and over) and median income in 2005 of all census families, and then subtracting the proportion of unemployed (unemployed people aged 15 and over as a percentage of people aged 15 and over who were in the labor force). The dissemination areas where the children resided were then classified into low, medium, or high SES based upon a tertile split

#### Body Mass Index (BMI)

When children attended their appointment, height and weight were measured by a trained health assistant and BMI (kg/m^2^) was calculated for each participant.

#### Physical Activity

Children's physical activity was assessed through a proxy report completed by a parent. The questionnaire was a modified version of the Children's Leisure Activities Study Survey (CLASS) [32]. It consisted of a checklist of 9 physical activities and parents indicated how many times (frequency), and the average minutes each time (duration) their child participated in an activity during the weekday and/or weekend of a typical week. Weighted means for weekdays and weekends were used to calculate the total weekly physical activity minutes per participant. The CLASS questionnaire has shown good reliability in 5-6 years olds with test re-test (percentage of agreement) ranging from 62% to 94% [32].

#### Physical Activity Concerns

Parents were asked to identify any conditions or diseases that may limit their child's ability to engage in physical activity, (e.g., "Does your child have any problems that would hinder them from doing physical activities?) If yes, parents were asked to record the difficulty. These difficulties were classified into five main categories, 84 (5.1%) participants had asthma/allergies, 17 (1.0%) had a motor skill delay/issue, 4 (0.2%) had a heart/lung condition, 3 (0.2%) had type 1 diabetes, and 34 (2.1%) had an "other" condition. This variable was coded, "0" for no and "1" for yes.

#### Seasons

The month in which the parents completed the proxy report was used to classify the child into a season. According to a recent review [33], most studies based in the northern hemisphere have classified seasons as: spring (March to May); summer (June to August); autumn/fall (September to November); and, winter (December to February). This is consistent with the seasons found in Edmonton. Three dummy variables were created with winter as the reference.

#### Daycare

Parents were asked, "Does your child attend any of the following: day care, play school, preschool, or kindergarten?" This variable was coded, "0" for no and "1" for yes.

### Data Analysis

Analyses were completing using SAS version 9.2 [SAS Institute Inc., Cary, NC]. Normality of distributions was examined for outcome variables through inspections of the normal probability plots. The outcome variables screen time and TV/movie were normally distributed, but the video game and computer variables were highly positively skewed. Descriptive statistics were calculated, including average screen time and TV/movie weekly minutes by SES group and prevalence of computer and video game users by SES groups. This was followed by a linear trend analysis for screen time across SES groups. Also, dependent sample t-tests and Wilcoxon Signed Rank tests were conducted to compare weekday versus weekend screen time minutes.

Multiple linear regression models were then conducted for weekly screen time and TV/movie minutes. Since a small proportion of the sample engaged in computer and video games these variables were categorized into "users" and "non-users" and multiple logistic regression models were conducted. High SES was the reference group for both linear and logistic regression models. A number of studies have found that correlates of sedentary behavior [5,34,35], and the impact of neighborhood environment differ between boys and girls [22,36-41], so gender by neighborhood SES interaction variables were tested in all models. Since power for moderation analyses is generally low, the significance level was set to alpha = .10 for these analyses [42]. Also based on priori assumptions of confounding [43] as well as previous literature on screen time [17,35] and neighborhood SES [22] the variables age, day care status, physical activity concerns, seasonal variations, BMI, and physical activity were examined as a potential confounders. A backward elimination procedure, with a cut-off of *p *≤ 0.15, was used to identify key confounders for each model after the two SES dummy variables were forced in. Before proceeding with our analyses, we examined whether any differences existed between the included and missing cases on some key variables. No significant differences existed for screen time, *χ*^2 ^(1) = 0.06, *p *= 0.81, or any of the subscales of screen time between the included and missing cases. Sample sizes of *n *= 805 and *n *= 828 were deemed sufficient to detect medium to small associations at an alpha level of 0.05 [44].

## Results

Descriptive data for the sample are presented in Table 1. The overall mean (standard deviation) weekly minutes for screen time was 834.1 (493.2) and for TV/video 678 (414.18). The overall median (interquartile range) weekly minutes for video games was 0 (13) and for computers 60 (150). Participants engaged in significantly more (*p *< 0.01) screen time, TV/movie, video game, and computer minutes on weekends compared to weekdays. Also, boys engaged in significantly more (*p *< 0.01) overall weekly screen time and video game minutes compared to girls. As well, 42% of participants engaged in more than 2 hours of screen time (40% for girls and 45% for boys) and 78% percent of participants engaged in more than 1 hour of screen time (68% for girls and 80% for boys).

**Table 1:** Participant information.

Characteristic		Boys *n = 828*	Girls *n *= 805	Overall *n *= 1633
Age	4 years (%) (5)	45.4	52.2	48.7
	5 years (%)	54.6	47.8	51.3
PA Concerns	Yes (%)	10.3	6.2	8.3
	No (%)	89.7	93.8	91.7
Day Care	Yes (%)	87.7	86.6	87.2
	No (%)	12.2	13.4	12.8
Seasons	Fall (%)	15.5	15.4	15.4
	Winter (%)	17.5	13.0	15.3
	Spring (%)	28.9	29.6	29.2
	Summer (%)	38.1	42.0	40.1
SES	Low (%)	21.4	22.0	21.7
	Medium (%)	35.6	35.7	35.6
	High (%)	43.0	42.4	42.7
PA (minutes/week)		708.1 (512.6)	657.3 (511.3)	683.0 (512.5)
BMI (kg/m^2^)		16.1 (2.3)	16.0 (2.2)	16.0 (2.2)

Data presented as mean +/- standard deviations or %.

SES = Socioeconomic Status; PA = Physical Activity; BMI = Body Mass Index

For girls, a significant decreasing trend for average minutes of overall screen time (P_trend _< 0.01) and TV/movie (P_trend _= 0.01) weekly minutes across SES groups was observed (see Table 2). For all children, a significant decreasing trend for video game users (P_trend _< 0.01) and significant increasing trend for computer users (P_trend _< 0.01) across SES groups was observed (see Table 2). Significant gender by neighborhood SES (low vs. high) interactions existed for overall screen time (*p *= 0.07) and TV/movie (*p *= 0.07) weekly minutes in the linear regression models. Thus, we stratified our analysis by gender and interpreted the results separately. After adjustment for key confounders in the linear regression models, girls living in low SES neighborhoods engaged in significantly more overall screen time and TV/movie weekly minutes compared to girls living in high SES neighborhoods (see Table 3). However, no statistically significant differences in overall screen time and TV/movie weekly minutes were observed between girls living in medium and high SES neighborhoods. Also, no statistically significant differences existed in overall screen time and TV/movie weekly minutes among boys living in low and high or medium and high SES neighborhoods.

**Table 2:** Mean (standard deviation) weekly minutes for total screen time and TV/movie, and proportions of users for video games and computer per SES category.

Characteristics	Low SES	Medium SES	High SES	
	Mean (SD)	Mean (SD)	Mean (SD)	Linear Test for Trend
Screen Time				
Boys	883.8 (555.7)	871.6 (491.8)	837.8 (452.0)	*F *(1) = 1.26, *p *= 0.26
Girls	906.2 (556.8)	810.1 (512.8)	756.7 (440.5)	*F *(1) = 9.90, *p *< 0.01
Overall	894.2 (555.5)	839.8 (503.3)	798.2 (448.0)	*F *(1) = 9.03, *p *< 0.01
TV/Movie				
Boys	660.9 (409.5)	688.1 (416.0)	664.73 (370.7)	*F *(1) = 0.00, *p *= 0.95
Girls	739.8 (453.2)	701.3 (451.1)	644.7 (403.1)	*F *(1) = 6.00, *p *= 0.01
Overall	697.5 (431.5)	694.9 (434.2)	654.96 (386.72)	*F *(1) = 3.22, *p *= 0.07
	(%)	(%)	(%)	Linear Test for Trend
Video Games				
Boys	43.3	36.1	32.0	*Z *(1) = -2.60, *p *< 0.01
Girls	18.5	15.4	11.2	*Z *(1) = -2.32, *p *= 0.02
Overall	31.8	25.4	21.8	*Z *(1) = -3.47, *p *< 0.01
Computer				
Boys	52.4	61.3	65.9	*Z *(1) = 3.01, *p *< 0.01
Girls	56.2	56.3	66.5	*Z *(1) = 2.60, *p *< 0.01
Overall	54.2	58.7	66.2	*Z *(1) = 3.97, *p *< 0.01

SES = Neighborhood socioeconomic status

**Table 3:** Gender-specific unadjusted and adjusted linear regression models predicting weekly total screen time and TV/movie minutes.

Neighborhood SES	Unadjusted		Adjusted ^a^	
**Boys**	**β**	**95%CI**	**β**	**95%CI**

Screen Time (Mins/Week)				
Low	45.97	-40.53 to 132.46	28.84	-57.99 to 115.67
Medium	33.78	-43.09 to 110.65	23.81	-52.91 to 100.52
High	Reference		Reference	
TV/Movie (Mins/Week)				
Low	-3.86	-73.56 to 65.84	-15.07	-85.24 to 55.11
Medium	23.36	-38.58 to 85.31	15.05	-47.06 to 77.16
High	Reference		Reference	

**Girls**	**β**	**95%CI**	**β**	**95%CI**

Screen Time (Mins/Week)				
Low	149.50*	57.61 to 241.40	142.30*	49.75 to 234.85
Medium	53.39	-23.20 to 129.98	48.81	-28.09 to 125.70
High	Reference		Reference	
TV/Movie (Mins/Week)				
Low	95.04*	14.55 to 175.54	97.22*	16.63 to 177.81
Medium	56.58	-10.51 to 123.67	55.74	-11.36 to 122.85
High	Reference		Reference	

SES = Socioeconomic Status.

Regression coefficients are interpreted as the difference in screen time (mins/week) from the reference category.

^a ^The boys screen time model was adjusted for age, day care, BMI, the boys TV/movie model was adjusted for day care physical activity concerns, and BMI. The girls screen time model was adjusted for day care, and the girls TV/movie model was adjusted for physical activity concerns.

* *p *< .05.

No statistically significant gender by neighborhood SES (low vs. high) interactions were observed for computers (*p *= 0.29) or video games (*p *= 0.51) in the logistic regression models. Therefore we did not consider gender an effect modifier and ran the logistic regression models on the combined girls and boys sample. After adjustment for key confounders we found children living in low SES neighborhoods were significantly more likely (70%) to be video game users and less likely (41%) to be computer users compared to children living in high SES neighborhoods (see Table 4). Also, children living in medium SES neighborhoods were significantly less likely (29%) to be computer users compared to children living in high SES neighborhoods. However, no significant differences existed in the odds of playing video games between children in medium and high SES neighborhoods.

**Table 4:** Unadjusted and adjusted logistic regression models predicting computer and video game use.

Neighborhood SES	Unadjusted		Adjusted ^a^	
**Video Game**	**OR**	**95%CI**	**OR**	**95%CI**

Low	1.67*	1.26 - 2.23	1.70*	1.26 - 2.29
Medium	1.22	0.94 - 1.58	1.28	0.98 - 1.67
High	1.00		1.00	

**Computer**	**OR**	**95%CI**	**OR**	**95%CI**

Low	0.60*	0.47 - 0.79	0.59*	0.45 - 0.76
Medium	0.73*	0.58 - 0.91	0.71*	0.56 - 0.89
High	1.00		1.00	

SES = Socioeconomic Status.

^a ^The video game model was adjusted for gender, age, BMI. The computer model was adjusted for age, day care, physical activity concerns.

* *p *< .05.

## Discussion

We examined whether neighborhood SES was associated with screen time use among pre-school boys and girls in Edmonton, Canada after adjusting for various confounders. Girls living in low SES neighborhoods engaged in significantly more weekly screen time and TV/movie minutes compared to girls living in high SES neighborhoods. Children living in low SES neighborhoods were more likely to use video games and less likely to use computers compared to children living in high SES neighborhoods. Also, children living in medium SES neighborhoods were less likely to use computers compared to children living in high SES neighborhoods We also found a large portion of our sample exceeded the guidelines for screen time recommended by American and Canadian pediatric associations [7,8].

Consistent with other studies of older children we found screen time activities differed between boys and girls [5,34,35]. Even at a pre-school age boys engaged in more screen time weekly minutes than girls, especially in video games. As well, associations between overall screen time and TV/video weekly minutes with neighborhood SES were observed only among girls. Therefore future screen time and environment research among children should consider the moderating effects of gender as well as explore potential explanations for these effects.

Despite some gender differences in the association between neighborhood SES and screen time, there still appears to be an overall association with screen time and neighborhood SES. Apart from computer use, children residing in lower SES neighborhoods engaged in more screen time activities. The opposite association observed between neighborhood SES and computer use may be related to access. That is, families residing in lower SES neighborhoods may not be able to afford a home computer [45].

Our analysis did not include a measure of family SES, however neighborhood SES has found to be associated with TV viewing independent of family SES among youth [22]. Therefore we speculate on two possible mechanisms that may help to explain why children in low SES neighborhoods in our study engaged in more overall screen time, TV/movie, and video game weekly minutes. First, parental perceptions of poor neighborhood safety are thought to limit children's outdoor play and increase sedentary indoor activity such as screen time [1,22,25,26,38,46-50]. For example, a study in the United States found that pre-school children who lived in neighborhoods that their mothers perceived as unsafe viewed more TV [46]. Similarly, Canadian children in neighborhoods that were perceived as unsafe engaged in less outdoor, unstructured play and were more likely to stay indoors and participate in sedentary activities [50]. Second, higher SES neighborhoods which typically have more resources, recreation facilities, and play areas can offer more alternative activities to screen time for children [22,37-40,49,51-53]. For example, reduced access to facilities in lower SES block groups was associated with a decrease in physical activity and an increase in overweight in children [51]. Future research should explore these potential mechanisms through mediation analyses.

Most studies examining screen time behaviors among children have focused on TV viewing [54]. Therefore, along with the TV analysis a unique aspect of our study was the analysis of video games and computer use. TV/movie minutes were higher than video games and computers for both boys and girls. These numbers are consistent with two recent reviews [55,56]. Basically, TV remains the most dominant screen time behavior among young children [56]. However, this may change when children become older [56].

Strengths of the study include the large pre-school aged sample and the inclusion of video games and computer use analyses. Also, this was the first study to date to examine the associations between neighborhood SES and screen time among pre-school children. Limitations of the study include the cross sectional design and the use of parental reports with an unvalidated questionnaire for screen time. According to a recent systematic review the majority of studies measuring TV viewing in children and adolescents use parental reports and very few of the questionnaires have been psychometrically tested [57]. Therefore a need exists for more standardized approaches of measurement for screen time behavior among children. Finally, though we included neighborhood SES in our analyses, we would have preferred to also have an indication of household SES for our participants.

These findings raise some important questions regarding the neighborhood environment and its impact on health behavior in young children. If in fact neighborhood SES predicts screen time independent of family SES, then addressing issues such as neighborhood safety and limited access to facilities are complex issues that require political will and commitment of financial resources. These things also require coordinated action and effort across various levels and departments of government and other key stakeholders [25]. From a public health perspective, these findings have potential implications for interventions designed to reduce screen time among children. Interventions may need to consider environmental factors and be gender specific.

## Conclusions

Some consideration should be given to providing alternative activity opportunities for children, especially girls who live in low SES neighborhoods. Also, future research should continue to investigate the independent effects of neighborhood SES on screen time as well as potential mediating variables (e.g., neighborhood safety, neighborhood recreation facilities) for this relationship.

## Competing interests

The authors declare that they have no competing interests.

## Authors' contributions

VC participated in the background research and the design of the study. She performed the statistical analysis and drafted the manuscript. JCS conceived of the study, participated in its design, and coordination. He helped with statistical analysis and writing the manuscript. NC assisted with data analysis. LC contributed to the background research. All authors read and approved the final manuscript.

## Pre-publication history

The pre-publication history for this paper can be accessed here:

http://www.biomedcentral.com/1471-2458/10/367/prepub
